# Supplementary material for: Learning and forgetting using reinforced Bayesian change detection
Source: PLoS Comput Biol. 2019 Apr 17;15(4):e1006713. doi: 10.1371/journal.pcbi.1006713 (PMC6488101; doi:10.1371/journal.pcbi.1006713)
Supplement: S1 Text — (PDF) [file pcbi.1006713.s001.pdf]

Appendix A Normalizing constant of the exponential mixture prior distribution

Let us consider a prior distribution from the exponential family of the form

$$f(\mathbf{z}|\boldsymbol{\theta}) = h(\mathbf{z})\exp(\boldsymbol{\theta}\mathbf{T}(\mathbf{z}) - A(\boldsymbol{\theta})). \tag{1}$$

Assuming that the approximate posterior $q(\mathbf{z}|\boldsymbol{\theta}_{j-1})$ has the same form as the prior distribution (which arises naturally in the VB scheme we are using), we are interested in deriving

$$\begin{aligned}
Z(w,\boldsymbol{\theta}_{j-1},\boldsymbol{\theta}_0) &= \int f(\mathbf{z}|\boldsymbol{\theta}_{j-1})^w f(\mathbf{z}|\boldsymbol{\theta}_0)^{1-w} d\mathbf{z} \\
&= \int \exp\left(w\left(\boldsymbol{\theta}_{j-1}\mathbf{T}(\mathbf{z}) + \log h(\mathbf{z}) - A(\boldsymbol{\theta}_{j-1})\right) + \right. \\
&\qquad \left. (1-w)\left(\boldsymbol{\theta}_0\mathbf{T}(\mathbf{z}) + \log h(\mathbf{z}) - A(\boldsymbol{\theta}_0)\right)\right) dz \\
&= \int h(\mathbf{z}) \exp\left(w\left(\boldsymbol{\theta}_{j-1}\mathbf{T}(\mathbf{z}) - A(\boldsymbol{\theta}_{j-1})\right) + \right. \\
&\qquad \left. (1-w)\left(\boldsymbol{\theta}_0\mathbf{T}(\mathbf{z}) - A(\boldsymbol{\theta}_0)\right)\right) \\
&\quad \times \exp\left(A\left(w\boldsymbol{\theta}_{j-1} + (1-w)\boldsymbol{\theta}_0\right) - A\left(w\boldsymbol{\theta}_{j-1} + (1-w)\boldsymbol{\theta}_0\right)\right) d\mathbf{z} \\
&= \underbrace{\int h(\mathbf{z}) \exp\left(\left(w\boldsymbol{\theta}_{j-1} + (1-w)\boldsymbol{\theta}_0\right)\mathbf{T}(\mathbf{z}) - A\left(w\boldsymbol{\theta}_{j-1} + (1-w)\boldsymbol{\theta}_0\right)\right) d\mathbf{z}}_{=1} \\
&\quad \times \exp(-wA(\boldsymbol{\theta}_{j-1}) - (1-w)A(\boldsymbol{\theta}_0) + A\left(w\boldsymbol{\theta}_{j-1} + (1-w)\boldsymbol{\theta}_0\right)) \\
&= \exp(-wA(\boldsymbol{\theta}_{j-1}) - (1-w)A(\boldsymbol{\theta}_0) + A\left(w\boldsymbol{\theta}_{j-1} + (1-w)\boldsymbol{\theta}_0\right)).
\end{aligned} \tag{2}$$

This result simplifies when combined with the numerator of Eq 8:

$$\begin{aligned}
p(\mathbf{z}|\boldsymbol{\theta}_{j-1},\boldsymbol{\theta}_0,w) &= \frac{h(\mathbf{z})\exp\left(\left(w\boldsymbol{\theta}_{j-1} + (1-w)\boldsymbol{\theta}_0\right)\mathbf{T}(\mathbf{z}) - wA(\boldsymbol{\theta}_{j-1}) - (1-w)A(\boldsymbol{\theta}_0)\right)}{\exp(-wA(\boldsymbol{\theta}_{j-1}) - (1-w)A(\boldsymbol{\theta}_0) + A(w\boldsymbol{\theta}_{j-1} + (1-w)\boldsymbol{\theta}_0))} \\
&= h(\mathbf{z})\exp\left(\left(w\boldsymbol{\theta}_{j-1} + (1-w)\boldsymbol{\theta}_0\right)\mathbf{T}(\mathbf{z}) - A\left(w\boldsymbol{\theta}_{j-1} + (1-w)\boldsymbol{\theta}_0\right)\right)
\end{aligned} \tag{3}$$

which has the form of a distribution from the exponential family where the natural parameters of the two parts of the mixture are weighted according to w and $1-w$.

If we are to find an analytical form to the lower bound $\mathcal{L}(q(\mathbf{z},w)) = \mathbb{E}_{q(\mathbf{z},w)}\left[\log p(\mathbf{x},\mathbf{z},w) - \log q(\mathbf{z},w)\right]$, we also need to compute the expected log-partition function given the approximate posterior $q(\mathbf{z},w)$: $-\mathbb{E}_{q(\mathbf{z},w)}\left[A\left(w\boldsymbol{\theta}_{j-1} + (1-w)\boldsymbol{\theta}_0\right)\right]$. This expectation has usually not a closed-form expression, but it can be approximated efficiently if we consider its second order Taylor

expansion. Indeed,

$$
\begin{aligned}
A\left(w\boldsymbol{\theta}_{j-1}+(1-w)\boldsymbol{\theta}_0\right) = {} & A\left(w_0\boldsymbol{\theta}_{j-1}+(1-w_0)\boldsymbol{\theta}_0\right) \\
& + \nabla_{w_0} A\left(w_0\boldsymbol{\theta}_{j-1}+(1-w_0)\boldsymbol{\theta}_0\right)(w-w_0) \\
& + \frac{\nabla^2_{w_0} A\left(w_0\boldsymbol{\theta}_{j-1}+(1-w_0)\boldsymbol{\theta}_0\right)}{2}(w-w_0)^2 + \mathcal{O}\big(|w_0-w|^3\big).
\end{aligned} \tag{4}
$$

If we replace w_0 by the expected value of w under the approximate posterior $w_0 \triangleq \mathbb{E}_{q(w)}\left[w\right] = \widehat{w}$, and given that $\mathbb{E}_{q(w)}\left[(w-\widehat{w})\right] = 0$, the expected value of the log-partition function can be approximated with

$$
\begin{aligned}
\mathbb{E}_{q(w)}\left[A\left(w\boldsymbol{\theta}_{j-1}+(1-w)\boldsymbol{\theta}_0\right)\right] \approx {} & A\left(\widehat{w}\boldsymbol{\theta}_{j-1}+(1-\widehat{w})\boldsymbol{\theta}_0\right) \\
& + \frac{1}{2}\nabla^2_{\widehat{w}} A\left(\widehat{w}\boldsymbol{\theta}_{j-1}+(1-\widehat{w})\boldsymbol{\theta}_0\right)\mathbb{V}ar_{q(w)}[w] \\
\text{with } \widehat{w} = \mathbb{E}_{q(w)}\left[w\right].
\end{aligned} \tag{5}
$$
