# Supplementary material for: Learning and forgetting using reinforced Bayesian change detection
Source: PLoS Comput Biol. 2019 Apr 17;15(4):e1006713. doi: 10.1371/journal.pcbi.1006713 (PMC6488101; doi:10.1371/journal.pcbi.1006713)
Supplement: S2 Text — (PDF) [file pcbi.1006713.s002.pdf]

Appendix B HAFVF update equations correspondence in classical RL

The update equations of Eq 16 have a simple correspondence in classical frequentist Q-learning. Let us first consider the case of the mean update in Bayesian Q-Learning without adaptive forgetting where we have

$$\begin{aligned} \mu_j^\mu &= \frac{\kappa_{j-1}^\mu \mu_{j-1}^\mu + \mathbf{x}_j}{\kappa_{j-1}^\mu + 1} \\ &= (1-\eta)\mu_{j-1}^\mu + \eta x_j \\ &\quad \text{where } \eta = \frac{1}{\kappa_{j-1}^\mu + 1} \\ &= \mu_{j-1}^\mu + \eta \underbrace{\left(x_j - \mu_{j-1}^\mu\right)}_{\text{RPE}}. \end{aligned} \tag{6}$$

We can see that the Reward Prediction Error (RPE) of the Rescola-Wagner (RW) update emerges naturally in one considers that the learning rate η is equal to the inverse of the sum of the number of trials observed plus the prior observation belief κ_0^μ (meaning that the learning rate decreases each time an observation is made by a factor $\frac{\kappa_0^\mu + n}{\kappa_0^\mu + n + 1}$).

We can now look at the update of the expected value of the mean reward. It can be reformulated as

$$\begin{aligned} \mu_j^\mu &= \frac{\widehat{w}\kappa_{j-1}^\mu \mu_{j-1}^\mu + (1-\widehat{w})\kappa_0^\mu \mu_0^\mu + x_j}{\kappa_j^\mu} \\ &= \frac{\widehat{w}\kappa_{j-1}^\mu}{\kappa_j^\mu}\mu_{j-1}^\mu + \frac{(1-\widehat{w})\kappa_0^\mu}{\kappa_j^\mu}\mu_0^\mu + \frac{x_j}{\kappa_j^\mu}. \end{aligned}$$

Without loss of generality, we can consider that the prior mean reward is equal to 0, which simplifies the above equation:

$$\mu_j^\mu = \frac{\widehat{w}\kappa_{j-1}^\mu}{\kappa_j^\mu}\mu_{j-1}^\mu + \frac{x_j}{\kappa_j^\mu}.$$

One can easily see that, in this context, we can recover the classical RW update term:

$$\begin{aligned} \mu_j^\mu &= (1-\eta)\mu_{j-1}^\mu + \frac{\eta}{(1-\widehat{w})\kappa_{j-1}^\mu + 1} x_j \\ &= \mu_{j-1}^\mu + \eta \underbrace{\left(\frac{x_j}{(1-\widehat{w})\kappa_{j-1}^\mu + 1} - \mu_{j-1}^\mu\right)}_{\text{RPE}} \\ \text{where } \eta &= \frac{(1-\widehat{w})\kappa_{j-1}^\mu + 1}{\kappa_j^\mu}. \end{aligned}$$

The current observation x_j is decayed by a factor proportional to the product of the previous effective memory κ_{j-1} and the trust allowed to the prior $1-\widehat{w}$. This means that the agent learns less trials that need to be discarded (as they are more likely to be generated by the prior distribution than the previous posterior), especially when the agent has a high memory. This account for the fact that an accident is less likely after a long than a short steady training.
