# Supplementary material for: Learning and forgetting using reinforced Bayesian change detection
Source: PLoS Comput Biol. 2019 Apr 17;15(4):e1006713. doi: 10.1371/journal.pcbi.1006713 (PMC6488101; doi:10.1371/journal.pcbi.1006713)
Supplement: S3 Text — (PDF) [file pcbi.1006713.s003.pdf]

Appendix C Counterfactual learning update equations

C.1 Continuous Updating

We consider that the agent adopts some update policy based on a family of inferred update distributions $\widetilde{x}_{j+1} \sim f\left(\boldsymbol{\theta}_j(s,a), \boldsymbol{\theta}_0(s,a), \boldsymbol{\phi}_j, \boldsymbol{\phi}_0, \boldsymbol{\beta}_0\right)$. As our notation suggests, this update distribution is either a function of the previous approximate posterior $\boldsymbol{\theta}_j$, of the prior $\boldsymbol{\theta}_0$ or both.

If $\widetilde{x}_{j+1} \sim f(\boldsymbol{\theta}_j(s,a))$, we are in the presence of an optimistic limit case where the agent does not consider that the environment will change anymore when she has selected an action: once some $r_\tau(s,a)$ with $\tau \in 1:j$ is observed, the resulting posterior predictive distribution is used to update the variational parameters in the next trials until a is selected again.

Conversely, the pessimistic model of evolution of $\widetilde{x}_{j+1}$ considers that the upcoming values of $r(s,a)$ are extremely variable, and that the agent should not trust anything more than its broad, initial marginal prior $p(\widetilde{x}_{j+1} | \boldsymbol{\theta}_0)$.

A third case of update distribution can be used: the agent can actually consider that the new value of $\widetilde{x}_{j+1}$ would likely be drawn from a mixture of the prior and posterior approximate marginal distributions, similar to the distribution implemented in Eq 9 (and possibly Eq 13):

$$\begin{aligned} \widetilde{x}_{j+1} &\sim p(\widetilde{x}_{j+1} | \mathbf{z}) \\ \mathbf{z} &\sim p(\mathbf{z} | w(\boldsymbol{\theta}_j(s,a) - \boldsymbol{\theta}_0(s,a)) + \boldsymbol{\theta}_0). \end{aligned} \tag{7}$$

The next section details the update equation in these three cases.

C.2 Approximate posterior updating of non-selected actions

In order to develop the update equations of the variational parameters in the case of counterfactual learning, one must first derive an ELBO for this specific case. Recall that, if x_{j+1} were observed, the ELBO would have the form:

$$\mathcal{L}_{j+1}(q(\mu,\sigma,w,b)) = \mathbb{E}_{(\mu,\sigma,w,b)}\left[\log p(x_{j+1},\mu,\sigma,w,b|\boldsymbol{\theta}_{j+1},\boldsymbol{\theta}_0) - \log q(\mu,\sigma,w,b)\right].$$

In the case of an unobserved datapoint, we take the expected value of the ELBO under some model of the evolution of $x_{j+1} \sim f(\boldsymbol{\theta}_j, \boldsymbol{\theta}_0)$

$$\mathbb{E}_f\left[\mathcal{L}_{j+1}(q(\mu,\sigma,w,b))\right] = \mathbb{E}_f\left[\mathbb{E}_{(\mu,\sigma,w,b)}\left[\log p(x_{j+1},\mu,\sigma,w,b|\boldsymbol{\theta}_{j+1},\boldsymbol{\theta}_0) - \log q(\mu,\sigma,w,b)\right]\right].$$

One can easily see that the update of the variational parameters of the action not taken under the optimistic assumption that the environment will not change takes the form of:

$$\begin{aligned} \mu^\mu_{j+1} &= \frac{\widehat{w}\kappa^\mu_j\mu^\mu_j + (1-\widehat{w})\kappa^\mu_0\mu^\mu_0}{\kappa^\mu_{j+1}} + \frac{\widehat{x_{j+1}}}{\kappa^\mu_{j+1}} \\ \kappa^\mu_{j+1} &= \widehat{w}\kappa^\mu_j + (1-\widehat{w})\kappa^\mu_0 + 1 \\ \alpha^\sigma_{j+1} &= \widehat{w}\alpha^\sigma_j + (1-\widehat{w})\alpha^\sigma_0 + \frac{1}{2} \\ \beta^\sigma_{j+1} &= \widehat{w}\beta^\sigma_j + (1-\widehat{w})\beta^\sigma_0 + \\ &\quad \frac{1}{2}\left(\widehat{w}\kappa^\mu_{j+1}(\mu^\mu_{j+1}-\mu^\mu_j)^2 + (1-\widehat{w})\kappa^\mu_0(\mu^\mu_{j+1}-\mu^\mu_0)^2 + (\widehat{x_{j+1}}-\mu^\mu_{j+1})^2 + \mathbb{V}ar[x_{j+1}]\right) \end{aligned}$$

$$
\begin{aligned}
\text{where } \widehat{x_{j+1}} &= \mathbb{E}_q\left[\mathbb{E}_p\left[x_{j+1}\right]\right] \\
&= \mu_j^\mu \\
\text{and } \mathbb{V}ar[x_{j+1}] &= \mathbb{E}_q\left[\mathbb{E}_p\left[x_{j+1}^2\right]\right] - \mathbb{E}_q\left[\mathbb{E}_p\left[x_{j+1}\right]\right]^2 \\
&= \frac{\beta_j^\sigma}{\alpha_j^\sigma - 1} + \frac{\beta_j^\sigma}{\kappa_j^\mu(\alpha_j^\sigma - 1)}.
\end{aligned}
$$

Note that the posterior predictive variance of x_{j+1} is equal to the sum of the expected variance and the variance of the mean. A similar update paradigm can be used for the opposite limit case where it is assumed that the distribution of x_{j+1} is totally undetermined (i.e. that it has come back to the marginal prior distribution $p(x_j|\boldsymbol{\theta}_0)$):

$$
\begin{aligned}
\widehat{x_{j+1}} &= \mathbb{E}_q\left[\mathbb{E}_p\left[x_{j+1}\right]\right] \\
&= \mu_0^\mu \\
\mathbb{V}ar[x_{j+1}] &= \mathbb{E}_q\left[\mathbb{E}_p\left[x_{j+1}^2\right]\right] - \mathbb{E}_q\left[\mathbb{E}_p\left[x_{j+1}\right]\right]^2 \\
&= \frac{\beta_0^\sigma}{\alpha_0^\sigma - 1} + \frac{\beta_0^\sigma}{\kappa_0^\mu(\alpha_0^\sigma - 1)}.
\end{aligned}
$$

Finally, the mixed approach consist in considering that the value x_{j+1} is a weighted average of the two given the learned mixing coefficient:

$$
\begin{aligned}
\widehat{x_{j+1}} &= \mu_*^\mu \\
\mathbb{V}ar[x_{j+1}] &= \frac{\beta_*^\sigma}{\alpha_*^\sigma - 1} + \frac{\beta_*^\sigma}{\kappa_*^\mu(\alpha_*^\sigma - 1)} \\
&\text{where} \\
\mu_*^\mu &= \frac{\widehat{w}\kappa_j^\mu \mu_j^\mu + (1-\widehat{w})\kappa_0^\mu \mu_0^\mu}{\kappa_*^\mu} \\
\kappa_*^\mu &= \widehat{w}\kappa_j^\mu + (1-\widehat{w})\kappa_0^\mu \\
\alpha_*^\sigma &= \widehat{w}\alpha_j^\sigma + (1-\widehat{w})\alpha_0^\sigma \\
\beta_*^\sigma &= \widehat{w}\beta_j^\sigma + (1-\widehat{w})\beta_0^\sigma + \\
&\frac{1}{2}\left(\widehat{w}\kappa_j^\mu(\mu_*^\mu - \mu_j^\mu)^2 + (1-\widehat{w})\kappa_0^\mu(\mu_*^\mu - \mu_0^\mu)^2\right).
\end{aligned}
$$

Using this update scheme, the agent erases his memory of the posterior distribution at a rate dictated by the stability of the environment, and $\boldsymbol{\theta}_j \to \boldsymbol{\theta}_0$ as $j \to \infty$. Together with the decision algorithm presented in 2.6, this result shows that, as the posterior distributions broadens and approaches the initial prior, the likelihood of choosing this action will also increase because the expected random noise of the evidence accumulation process will also increase.

C.3 Delayed approximate posterior updating

Another approach for the agent to consider the evolution of the environment when selecting an action whose outcome has not been seen for a long time is to simulate the expected waning of the previous update across the elapsed interval of time.

Let us consider the case where the agent has chosen an action (e.g. left) at the trial j, and then the opposite action (right) for n trials. We assume that, during these n trials, she has been updating only the value of the right action, leaving the approximate posterior over the distribution parameters of the value of the left action untouched. When selecting left at the trial $j+n$, she considers that the weight of the posterior

component of the prior distribution has decreased exponentially for n trials. The prior then looks like:

$$\frac{p(\mathbf{z}|\boldsymbol{\theta}_{j-n})^{w^n} p(\mathbf{z}|\boldsymbol{\theta}_0)^{1-w^n}}{Z}$$

In order to compute the NCVMP update of the approximate posterior parameters over w, we then need to compute the expected value of w^n. If $q_j(w)$ is set to be a beta distribution with parameters $\boldsymbol{\phi}_j = \{\phi_{1j}, \phi_{2j}\}$, we have

$$\mathbb{E}_{q_j(w)}\left[w^n\right] = \frac{\Gamma(\phi_{2j})\Gamma(\phi_{1j}+n)}{B(\phi_{1j},\phi_{2j})\Gamma(\phi_{1j}+\omega_{2j}+n)} \quad (8)$$

where $B(a,b) = \frac{\Gamma(a)\Gamma(b)}{\Gamma(a+b)}$. The expected value of the squared decay w^{2n} (used to compute the variance in the expected value of the second order Taylor expansion of the log-partition function, see A) can be computed readily with the same formula by replacing n by $2n$.

Unlike the previous method, the shape parameter of the gamma distribution over the variance does not need to be greater than 1 here, as we do not need to simulate the variance of the non-selected action value. However, this is true only for the single stage-case, as the multi-stage case also bases its value updates on expected squared values of rewards, thereby requiring $\alpha_0^\sigma > 1$ too.
