# Supplementary material for: Learning and forgetting using reinforced Bayesian change detection
Source: PLoS Comput Biol. 2019 Apr 17;15(4):e1006713. doi: 10.1371/journal.pcbi.1006713 (PMC6488101; doi:10.1371/journal.pcbi.1006713)
Supplement: S4 Text — (PDF) [file pcbi.1006713.s004.pdf]

Appendix D Discount factor inference

D.1 Model specification

The main idea we will try to develop here is that the discount factor γ can be considered as a latent random variable, so that inference is made about its value in order to get the most precise estimate of the posterior predictive distribution of the long term return of an action $p(V(s,a)|x_{\leq j})$.

For practical reasons, it is easier to model separately the factorized distribution of the current, immediate state-action reward $p(r(s,a))$ and the discounted value of the future state $p(\gamma v(s'|s,a))$. In this notation, $\gamma v(s'|s,a)$ is a random variable representing the discounted long-term value of taking the action a in state s minus the value of the reward received while performing this action $r(s,a)$:

$$V(s,a) = r(s,a) + \gamma v(s'|s,a)$$

Following this logic, we define the following factorized distribution:

$$p\big(r(s,a), v(s'|s,a)\big) = p\big(r(s,a)|\mu^r(s,a), \sigma^r(s,a)\big)\, p\big(v(s'|s,a)|\mu^v(s,a), \sigma^v(s'|s,a), \gamma\big)$$

where $r(s,a)$ is normally distributed and $v(s'|s,a)$ is distributed according to the following modified Gaussian distribution:

$$p\left(v(s'|s,a)|\mu^v(s,a), \sigma^v(s,a), \gamma\right) = \gamma \frac{\exp\left\{\frac{\left(\mu^v(s,a) - \gamma v(s'|s,a)\right)^2}{2\sigma^v(s,a)^2}\right\}}{\sqrt{2\pi}\,\sigma^v(s,a)}.$$

It becomes clear that this distribution integrates to 1 over $v(s'|s,a)$, and that it corresponds to a Normal distribution over $\gamma v(s'|s,a)$. Therefore, the probability distribution of $V(s,a) = r(s,a) + \gamma v(s'|s,a)$ can be written as:

$$p(V(s,a)) = \mathcal{N}(\mu^r(s,a) + \mu^v(s,a),\, \sigma^r(s,a)^2 + \sigma^v(s,a)^2).$$

This joint probability distribution encodes, for each state-action pair the probability distribution of the associated long-term, discounted return.

We naturally define the prior probability of the discounting factor $\gamma \in [0;1]$ at $j = 1$ as a Beta distribution with parameters $\mathrm{B}(\alpha_0^\gamma, \beta_0^\gamma)$, and a $\mathcal{NG}^{-1}$ prior over the Gaussian parameters $\{\mu^r(s,a), \sigma^r(s,a), \mu^v(s,a), \sigma^v(s,a)\}$.

D.2 Learning from observed transitions and rewards

We now tackle the question of how to estimate the value of the state that has been reached $v(s'|s,a)$. Indeed, this value, contrary to the reward, is not directly observed but must be inferred from the state of arrival s'. We consider the following state-value inference model:

$$\begin{aligned} v(s'|s,a) &= \mathbb{E}_{\pi(a'|s')}\left[V(s',a')\right] \\ &= \mathbb{E}_{\pi(a'|s')}\left[r(s',a') + v(s'|s,a)\right]. \end{aligned} \tag{9}$$

Eq 9 stated that the agent assumes that the expected value of the next state is equal to the average of the various state-action values available, weighted by the probability of selection the corresponding action (the observed policy $\pi(a'|s')$). We assume that the agent does not have a direct access to the complete description of the policy (meaning that, whereas she can make a decision efficiently, she cannot retrieve the summary

statistics of this policy). This assumption is in accordance with the decision process described in 2.6. This policy can, however, be learned similarly to $r(s,a)$ using a multinomial distribution with a Dirichlet (or Beta in the case of Two-Alternative Forced Choice tasks) prior and approximate posterior: at each trial, the agent observes the action she has performed, and update her belief of the current policy.

Let us now consider how the expected log-joint probability of $v(s'|s,a)$ would appear in the ELBO if this value was observed under the mean-field assumption:

$$\mathbb{E}_{q_{j+1}(\mu^v(s,a),\sigma^v(s,a)^2,\gamma)}\left[\log p(v(s'|s,a)|\mu^v(s,a),\sigma^{v2},\gamma)\right] = -\frac{1}{2}\mathbb{E}_{q_{j+1}(\sigma^v(s,a)^2)}\left[\log \sigma^v(s,a)^2\right] - \frac{1}{2}\mathbb{E}_{q_{j+1}(\mu^v(s,a),\sigma^v(s,a)^2)}\left[\frac{(\gamma v(s'|s,a)-\mu^v(s,a))^2}{\sigma^v(s,a)^2}\right] + \mathbb{E}_{q_{j+1}(\gamma)}\left[\log\gamma\right] + \text{cst.} \tag{10}$$

Once the agent reaches the next state $\widehat{s'}$, inference of the value $\mathbb{E}\left[v(s'|s,a)|s'=\widehat{s'}\right]$ can be achieved by using the posterior predictive expectation of this formula:

$$\begin{aligned}
\mathbb{E}\left[v(s'|s,a)|s'=\widehat{s'}\right] &= \mathbb{E}_{p(v|x_{\leq j})}\left[v(\widehat{s'}|s,a)\right] \\
&\approx \mathbb{E}_{q_j(\mathbf{z})}\left[\mathbb{E}_{p(V,\pi|\mathbf{z})}\left[\mathbb{E}_{\pi}\left[V(\widehat{s'},a')\right]\right]\right] \\
&= \mathbb{E}_{q_j(\mathbf{z})}\left[\mathbb{E}_{p(r,v,\pi|\mathbf{z})}\left[\mathbb{E}_{\pi}\left[r(\widehat{s'},a')+\gamma v(s''|s',a')\right]\right]\right] \\
&= \mathbb{E}_{q_j(\mathbf{z})}\left[\mathbb{E}_{p(r,v,\pi|\mathbf{z})}\left[\sum_{a'\in A}\pi(a'|\widehat{s'})(r(\widehat{s'},a')+\gamma v(s''|\widehat{s'},a'))\right]\right] \\
&= \sum_{a'\in a_1,a_2}\frac{\delta_j^{\pi}(\widehat{s'},a')}{\sum_{a\in A}\delta_j^{\pi}(\widehat{s'},a)}(\mu_j^r(\widehat{s'},a')+\mu_j^v(\widehat{s'},a'))
\end{aligned} \tag{11}$$

where A is the set of actions available, and where we indexed with j each variational parameter to emphasize the fact that this expectation is made with respect to the previous posterior belief.

Eq 10 being linear and quadratic wrt $v(s'|s,a)$, we also need to solve the expected value $\mathbb{E}\left[v(s'|s,a)^2|s'=\widehat{s'}\right]$:

$$\mathbb{E}_{p\left(v|x_{\leq j}\right)}\left[v(s'|s,a)^2\right] \approx \mathbb{E}_{q_j(\mathbf{z})}\left[\mathbb{E}_{p(r,v,\pi|\mathbf{z})}\left[\Big(\sum_{a'\in A}\pi(a'|s')(r(s',a')+\gamma v(s''|s',a'))\Big)^2\right]\right]. \tag{12}$$

The expression in Eq 12 can be computed iteratively (Algorithm 1):

Algorithm 1: $\mathbb{E}_{p(v(s'|s,a)|x_{\leq j})}\left[v(s'|s,a)^2|s'=\widehat{s'}\right]$ iterative computation

input : Factorized approximate posterior distribution q_{j-1}, state reached at trial j $\widehat{s'}$

Results: Posterior predictive estimate $\nu = \mathbb{E}_{p(v(s'|s,a)|x_{\leq j})}\left[v(s'|s,a)^2|s'=\widehat{s'}\right]$

$\nu = 0$

for $a \in A$ **do**

  for $a' \in A$ **do**

    if $a == a'$ **then**

      $\nu \mathrel{+}= \mathbb{E}_{q_{j-1}}\left[\pi(a|s')^2\right] \times$ // Eq 17

        $\left(\mathbb{E}_{q_{j-1}}\left[\mathbb{E}_p\left[r(s',a)^2\right]\right] + \mathbb{E}_{q_{j-1}}\left[\mathbb{E}_p\left[(\gamma v(s''|s',a))^2\right]\right]\right.$

      // Eq 14,Eq 16

        $\left.+2\mathbb{E}_{q_{j-1}}\left[\mathbb{E}_p\left[r(s',a)\right]\right]\mathbb{E}_{q_{j-1}}\left[\mathbb{E}_p\left[\gamma v(s''|s',a)\right]\right]\right)$ // Eq 13,Eq 15

    else

      $\nu \mathrel{+}= \mathbb{E}_{q_{j-1}}\left[\pi(a|s')\pi(a'|s')\right] \times$ // Eq 18

        $(\mathbb{E}_p\left[r(s',a)\right] + \mathbb{E}_{q_{j-1}}\left[\mathbb{E}_p\left[\gamma v(s''|s',a)\right]\right])\times$ // Eq 13,Eq 15

        $(\mathbb{E}_p\left[r(s',a')\right] + \mathbb{E}_{q_{j-1}}\left[\mathbb{E}_p\left[\gamma v(s''|s',a')\right]\right])$ // Eq 13,Eq 15

  end

end

using the following equivalences

$$\mathbb{E}_{q_{j-1}}\left[\mathbb{E}_{p(r|\mu^r,\sigma^r)}\left[r\right]\right] = \mu^r_{j-1} \tag{13}$$

$$\mathbb{E}_{q_{j-1}}\left[\mathbb{E}_{p(r|\mu^r,\sigma^r)}\left[r^2\right]\right] = {\mu^r_{j-1}}^2 + \frac{\beta^r_{j-1}}{(\alpha^r_{j-1}-1)\kappa^r_{j-1}} + \frac{\beta^r_{j-1}}{(\alpha^r_{j-1}-1)} \tag{14}$$

$$\mathbb{E}_{q_{j-1}}\left[\mathbb{E}_{p(v|\mu^v,\sigma^v,\gamma)}\left[\gamma v\right]\right] = \mu^v_{j-1} \tag{15}$$

$$\mathbb{E}_{q_{j-1}}\left[\mathbb{E}_{p(v|\mu^v,\sigma^v,\gamma)}\left[(\gamma v)^2\right]\right] = {\mu^v_{j-1}}^2 + {\sigma^v_{j-1}}^2 \tag{16}$$

$$\mathbb{E}_{q_{j-1}}\left[\pi(a|s)^2\right] = \left(\frac{\delta^{\pi(a|s)}_{j-1}}{\delta_0}\right)^2 + \frac{\delta^{\pi(a|s)}_{j-1}(\delta_0 - \delta^{\pi(a|s)}_{j-1})}{\delta_0^2(\delta_0+1)} \tag{17}$$

$$\mathbb{E}_{q_{j-1}}\left[\pi(a|s)\pi(a'|s)\right] = \frac{\delta^{\pi(a|s)}_{j-1}\delta^{\pi(a'|s)}_{j-1}}{\delta_0^2} - \frac{\delta^{\pi(a|s)}_{j-1}\delta^{\pi(a'|s)}_{j-1}}{\delta_0^2(\delta_0+1)} \tag{18}$$

$$\text{with } \delta_0 = \sum_{a' \in A} \delta^{\pi(a'|s)}$$

where we have dropped several state-action indices for clarity.

We can now take the expectation of Eq 10 under Eq 11 and Eq 12.

This formula has the advantage that the learning of the current distribution of $V(s,a)$ takes into account the uncertainty about the policy, about the reward distribution at the next trial and about the long-term discounted return of the actions in the next state.
