# Supplementary material for: Learning and forgetting using reinforced Bayesian change detection
Source: PLoS Comput Biol. 2019 Apr 17;15(4):e1006713. doi: 10.1371/journal.pcbi.1006713 (PMC6488101; doi:10.1371/journal.pcbi.1006713)
Supplement: S5 Text — (PDF) [file pcbi.1006713.s005.pdf]

Appendix E VPI

VPI [1–5] solves this problem by giving a positive bonus to each action value corresponding to the potential payoff of selecting this action given the current uncertainty about its value distribution. Formally, this bonus is computed as the posterior expectation of the gain of performing an action given the belief we have of the value taken by the other actions:

$$VPI(a) = \int p(\mu(a)|\mathbf{x}_{<j}) Gain_a(\mu(a))\, d\,\mu(a)$$
$$\text{where } Gain_a(\mu(a)) = \begin{cases} \mathbb{E}\left[\mu(a_2)\right] - \mu(a) & \text{if } a = a_1 \text{ and } \mu(a) < \mathbb{E}\left[\mu(a_2)\right] \\ -\mathbb{E}\left[\mu(a_1)\right] + \mu(a) & \text{if } a \neq a_1 \text{ and } \mu(a) > \mathbb{E}\left[\mu(a_1)\right] \\ 0 & \text{otherwise} \end{cases} \tag{19}$$

and where we have used the convention that a_1 and a_2 are the actions with the highest and second highest reward respectively. Eq E shows that the bonus of a given action is proportional to the expected gain of discovering that this action leads to a higher reward than all the others when it was thought to be sub-optimal, plus the expected gain of discovering that this action is sub-optimal when it was thought to be optimal.

Interestingly, low threshold of the NIGDM 2.6 favor choices that would have been also favored by the VPI approach: if an action has currently the highest estimate, it will be more encouraged if its variance is wide than if it is narrow, and conversely for punished action with a high variance.

Moreover, the evidence accumulation process can be enriched to incorporate the expected gain of performing an action: as the agent samples the means of the two action values given its current belief, she can add the difference in the gain bonuses computed as in Eq E. The resulting process can still be modelled as a Wiener process using the Stochastic Gradient Variational Bayes approach described in Sec 2.7.

A final point to consider is that VPI might be used to refine the expectation that a change of contingency has occured. In order to do so, the gain would need to incorporate the volatility measure. Limiting ourselves to a single forgetting layer, we would have a variational approximation to the Gain and VPI that would read:

$$Gain_a(\mu, w) = \begin{cases} \mathbb{E}_q[\mu_2 \mid w] - \mu(a) & \text{if } a = a_1 \text{ and } \mathbb{E}_q[\mu_2 \mid w] > \mu(a) \\ \mu(a) - \mathbb{E}_q[\mu_1 \mid w] & \text{if } a \neq a_1 \text{ and } \mathbb{E}_q[\mu_1 \mid w] < \mu(a) \\ 0 & \text{otherwise.} \end{cases}$$
$$VPI(a) = \iint q_j(\mu(a), w) Gain_a(\mu(a), w)\, d\,\mu(a)\, w$$

We can see that this formula involves the conditional expectancy of $\mu(a)$ given w, which is equal to $\mathbb{E}_{q_j}\left[\mu(a)\right]$ when the mean-field assumption is used. In other words, modelling the posterior covariance matrix of the HAFVF could lead to a exploration policy that would be guided by the uncertainty about the volatility of the environment.
