# Supplementary material for: Learning and forgetting using reinforced Bayesian change detection
Source: PLoS Comput Biol. 2019 Apr 17;15(4):e1006713. doi: 10.1371/journal.pcbi.1006713 (PMC6488101; doi:10.1371/journal.pcbi.1006713)
Supplement: S6 Text — (PDF) [file pcbi.1006713.s006.pdf]

Appendix F NIGDM properties

F.1 Discrete convergence property

Importantly, as for every Monte Carlo estimate, this estimate becomes more accurate (i.e. less entropic) as the expected number of iterations (absorption time) grows. In the previous example, this happens when the distance between the absorbing boundaries (threshold) is larger, in which case the agent is more likely to select the optimal option. To see this, consider the case where $p\left(r(a_1)|\mathbf{x}_{<j}\right)$ and $p\left(r(a_2)|\mathbf{x}_{<j}\right)$ are both Gaussian distributions with a known mean and variance, and where $\mu(a_1) > \mu(a_2)$. We then introduce the following result:

Lemma 1 *The probability of hitting the upper boundary ζ tends to 1 as the threshold grows, and the average displacement $\widehat{\delta z}$ concomitantly approaches the true probability that a_1 is more rewarded than a_2:*
$\widehat{\delta z} = \mathbb{E}_{p(r(a_1)|\mathbf{x}_{<j}),p(r(a_2)|\mathbf{x}_{<j})}\left[z - z_0\right] \rightarrow p(r(a_1) > r(a_2)|\mathbf{x}_{<j})$.

F.2 Continuous convergence property

The DDM has interesting properties that make it a good candidate to model decision making in Bayesian Reinforcement Learning. We first introduce the following result, which is a generalization of Lemma 1 to the continuous case:

Proposition 1 *If the DDM parameters (threshold ζ, relative start point $\nu \triangleq z_0/\zeta$, drift rate ξ and noise ς) are fixed within and between trials, the probability of hitting the boundary pointed by the drift approaches 1 as the distance between the boundaries grows.*

To show this, one can first start by showing that the Error Rate probability

$$P = \frac{\exp(-2\xi\zeta\nu/\varsigma^2) - \exp(-2\xi\zeta/\varsigma^2)}{1 - \exp(-2\xi\zeta/\varsigma^2)} \tag{20}$$

is monotonically increasing wrt ξ. This can be easily seen from the differential equation of this formula. Next, since $\xi \frac{dP}{d\xi} = \zeta \frac{dP}{d\zeta}$, and since $\frac{dP}{d\xi} > 0 \, \forall \xi \in \nabla$, then
$\operatorname{sign}\left(\frac{dP}{d\zeta}\right) = \operatorname{sign}(\xi) \, \forall \xi \in \nabla,\ \zeta \in \nabla^+$ and, therefore, P is monotonically increasing wrt ζ if $\xi > 0$ and decreasing if $\xi < 0$.

F.3 NIGDM similarity to QS

In order to show that the NIGDM mimics a QS algorithm for high thresholds, we need to consider example choices and reaction times generated by this process.

To this end, we first introduce an important result:

Proposition 2 *If a data collection is generated according to a classical Wiener Diffusion process where the drift and squared noise are sampled* ***at each trial*** *following some distribution $\tilde{\xi}, \tilde{\varsigma}^2 \sim p(\xi, \varsigma^2|\boldsymbol{\theta}_j)$, then this dataset cannot be distinguished from a dataset where the drift and noise are sampled similarly* ***within*** *trials.*

This can be proven trivially by considering the moment generating function of these two distributions. For the trial-by-trial process, we have:

$$\mathbb{E}\left[\mathrm{t}, a|\xi_j, \varsigma_j, \zeta\right] = \sum_{i \in \{1,2\}} e^{l a_i} \int e^{l\mathrm{t}} p(\mathrm{t}, a_i|\xi_j, \varsigma_j, \zeta, \tau, \nu) d\,\mathrm{t}\Big|_{l=1} \tag{21}$$

where t and a are the reaction time and choice, respectively. Now, as ξ and ς are unknown to us, we marginalize over their prior distribution (i.e. the current approximate posterior of $\boldsymbol{\theta}_j$):

$$\mathbb{E}\left[\mathrm{t}, a \,\middle|\, \boldsymbol{\theta}_j, \zeta\right] = \sum_{i \in \{1,2\}} e^{l c_i} \int e^{l\mathrm{t}} \iint p\left(\mathrm{t}, a_i \,|\zeta, \xi_j, \varsigma_j\right) p\left(\xi_j, \varsigma_j^2 | \boldsymbol{\theta}_j\right) dv_j \, d\varsigma_j^2 \, d\mathrm{t}\Big|_{l=1}.$$

One can see that this expression can be re-arranged into the moment generating function of the within-trial process. Because the two distributions have the same moments and the moment generating function exist for the DDM [6, 7], the two distributions are equal [8].

Proposition 2 is important because it states that, if we are able to generate from the NIGDM or fit this probability distribution to a dataset under the assumption of a between-trial variability, then this fit will also be valid in the case of a within-trial variability.

We will show in Sec 2.7 how this model can be optimized in a Maximum Likelihood and a Bayesian context.

We can now use Proposition 1 and Proposition 2 to show that this process does not automatically hit the current best estimate when the boundaries are distant enough from each other. During some trials, the difference between the sampled means $\widetilde{\mu}_1$ and $\widetilde{\mu}_2$ will have a sign opposite to the sign of the current estimate of $\mu_1^\mu - \mu_2^\mu$, and the agent will choose the action with the lowest value. More formally:

Proposition 3 *Under the NIGDM, the proportion of choices $p(a_1)$ tends to the posterior predictive probability $p(\mu_1 > \mu_2 | \mathbf{x}_{<j})$ as the threshold tends to infinity.*

This can be shown by considering Proposition 2 and Proposition 1 in the fixed-parameters case, and then considering that the expected probability of hitting the positive (or similarly the negative) boundary has the following distribution when ζ tends to infinity:

$$\begin{aligned}
\lim_{\zeta \to \infty} \mathbb{E}\left[p(a_1) | \boldsymbol{\theta}_j, \zeta\right] = &\iiiint \mathbb{1}\left[\mathrm{sign}(\mu_1 - \mu_2) = +1\right] \times \qquad (22) \\
&\quad p\left(\mu_1, \mu_2, \sigma_1, \sigma_2 | \boldsymbol{\theta}_j\right) \, d\mu_1 \, d\mu_2 \, d\sigma_1^2 \, d\sigma_2^2 \\
&= p(\mu_1 > \mu_2 | \boldsymbol{\theta}_j) \\
&\text{where } \boldsymbol{\theta}_j = \{\boldsymbol{\mu}^\mu, \boldsymbol{\kappa}^\mu, \boldsymbol{\alpha}^\sigma, \boldsymbol{\beta}^\sigma\} \qquad (23)
\end{aligned}$$

where $\mathbb{1}[x]$ is an operator that takes the value of 1 if its condition x is met and zero otherwise.
