# Supplementary material for: Learning and forgetting using reinforced Bayesian change detection
Source: PLoS Comput Biol. 2019 Apr 17;15(4):e1006713. doi: 10.1371/journal.pcbi.1006713 (PMC6488101; doi:10.1371/journal.pcbi.1006713)
Supplement: S7 Text — (PDF) [file pcbi.1006713.s007.pdf]

Appendix G Sampled Drift Optimisation

Under the assumption that decisions are made according to the scheme described in 2.6, the HAFVF can be optimized using a simple, informative model. Indeed, we can express the First Passage Time probability density function (and similarly the cumulative distribution function) as

$$p(\mathbf{y}_j|\boldsymbol{\theta}_j, \zeta, \nu, \tau) = \int p(\mathbf{y}_j|a_j, \mu_j, w_j, \tau_j, \sigma_j^2)\, p(\mu_j, \sigma_j^2|\boldsymbol{\theta}_j) d\mu_j, \sigma_j^2. \tag{24}$$

The central idea of the Sampled Drift Optimisation technique is to use the joint distribution (and not the marginal) in a VB framework. Considering that $\boldsymbol{\theta}_j = f(\boldsymbol{\theta}_0, \mathbf{x}_{<j})$, we can write the posterior probability of the DDM parameters at the trial j as:

$$p(\boldsymbol{\theta}_0, \mu_j, \sigma_j^2|\mathbf{y}_j, \zeta, \nu, \tau) = \frac{p(\mathbf{y}_j|\zeta, \nu, \tau, \mu_j, \sigma_j^2) p(\mu_j, \sigma_j^2|f(\boldsymbol{\theta}_0, \mathbf{x}_{<j}))}{p(\mathbf{y}_j, \zeta, \nu, \tau)}. \tag{25}$$

Let us now consider the posterior predictive probability density of the difference between the value of two action values at a specific trial. In the case of a single step task (i.e. without temporal discounting), the posterior predictive distribution of the reward difference is:

$$\begin{aligned}
p(R_1 - R_2|x_{1:j-1}) &\approx \int p(R_1 - R_2|\mathbf{z}_j) q(\mathbf{z}_j|\boldsymbol{\theta}_{j-1}) d\mathbf{z}_j \\
&\quad \text{with } R_i = r(s, a_i), \qquad \mathbf{z}_j = \{\mu_1^r, \mu_2^r, {\sigma_1^r}^2, {\sigma_2^r}^2\} \\
&= \iint p(\phi + R_2|\mu_1^r, {\sigma_1^r}^2) p(R_2|\mu_2^r, {\sigma_2^r}^2) dR_2\, q(\mathbf{z}_j|\boldsymbol{\theta}_{j-1}) d\mathbf{z}_j. \\
&\quad \text{where } \phi = R_1 - R_2 \\
&= \int p(\phi|\mu_1^r - \mu_2^r, {\sigma_1^r}^2 + {\sigma_2^r}^2) q(\mathbf{z}_j|\boldsymbol{\theta}_{j-1}) d\mathbf{z}_j
\end{aligned} \tag{26}$$

We see that we can express the posterior predictive probability of the difference of the Reward values as a normal distribution of mean $\mu^r - \mu^r$ and standard deviation $\sqrt{\sigma^{r2} + \sigma^{r2}}$, marginalized over these parameters given their posterior value at the present trial. We can plug the result of 26 into 24 and then into 25 to get:

$$p(\boldsymbol{\theta}_0|\mathbf{y}_j) = \frac{p(\mathbf{y}_j|\zeta, \nu, \tau, \mathbf{z}_j) p(\mathbf{z}_j|f(\boldsymbol{\theta}_0, \mathbf{x}_{<j}) p(\zeta, \nu, \tau)}{p(\mathbf{y}_j)}. \tag{27}$$

This model is easy to optimize using SGVB provided that we can estimate the Jacobian $\nabla_{\boldsymbol{\theta}_0} f(\boldsymbol{\theta}_0, \mathbf{x}_{<j})$.

It can also be applied to the discounted value $V(s,a)$ in multistep tasks:

$$\begin{aligned}
p\left(V(s,a) - V(s,a')|x_{1:j-1}\right) &\approx \int p\left(V(s,a) - V(s,a')|\mathbf{z}_j\right) q(\mathbf{z}_j|\boldsymbol{\theta}_{j-1}) d\mathbf{z}_j \\
&= \iint p\left(\phi + V(s,a')|\mu_1^R + \mu_1^v, {\sigma_1^R}^2 + {\sigma_1^v}^2\right) \times \\
&\quad p\left(V(s,a')|\mu_2^R + \mu_2^v, {\sigma_2^R}^2 + {\sigma_2^v}^2\right) dr(s,a')\, q(\mathbf{z}_j|\boldsymbol{\theta}_{j-1}) d\mathbf{z}_j \quad . \\
&\quad \text{where } \phi = V(s,a) - V(s,a') \\
&= \int p\left(\phi \Big| \sum_{u \in \{r,v\}} \mu_1^u - \mu_2^u, \sum_{u \in \{r,v\}} \sum_{a \in A} {\sigma_a^u}^2\right) q(\mathbf{z}_j|\boldsymbol{\theta}_{j-1}) d\mathbf{z}_j
\end{aligned} \tag{28}$$

References

1. Dearden R, Friedman N, Russell S. Bayesian Q-Learning. In: American Association of Artificial Intelligence (AAAI)-98; 1998. p. 761–768.

2. Dearden R, Dearden R, Friedman N, Friedman N, Andre D, Andre D. Model based Bayesian exploration. Proceedings of the fifteenth Conference on Uncertainty in Artificial Intelligence. 1999;(Howard 1966):150–159.

3. Keramati M, Dezfouli A, Piray P. Speed/accuracy trade-off between the habitual and the goal-directed processes. PLoS computational biology. 2011;7(5):e1002055. doi:10.1371/journal.pcbi.1002055.

4. Mcallister R, Dziugaite K. Bayesian Reinforcement Learning. 2013;35(March):1–21.

5. Viejo G, Khamassi M, Brovelli A, Girard B. Modelling choice and reaction time during instrumental learning through the coordination of adaptive working memory and reinforcement learning. Fourth Symposium on Biology of Decision - Making (SBDM 2014). 2014;9(August). doi:10.3389/fnbeh.2015.00225.

6. Grasman RPPP, Wagenmakers EJ, Van Der Maas HLJ. On the mean and variance of response times under the diffusion model with an application to parameter estimation. Journal of Mathematical Psychology. 2009;53(2):55–68. doi:10.1016/j.jmp.2009.01.006.

7. Wagenmakers EJ, Grasman RPPP, Molenaar PCM. On the relation between the mean and the variance of a diffusion model response time distribution. Journal of Mathematical Psychology. 2005;49:195–204. doi:10.1016/j.jmp.2005.02.003.

8. Grimmett G, Welsh D. Probability, An Introduction. 2nd ed. Oxford University Press; 1986. Available from: http://www.amazon.ca/exec/obidos/redirect?tag=citeulike09-20&path=ASIN/0198503687.
